# Dental Implants with Different Neck Design: A Prospective Clinical Comparative Study with 2-Year Follow-Up

**DOI:** 10.3390/ma13051029

**Published:** 2020-02-25

**Authors:** Pietro Montemezzi, Francesco Ferrini, Giuseppe Pantaleo, Enrico Gherlone, Paolo Capparè

**Affiliations:** 1 Dental School, Vita-Salute San Raffaele University, 20132 Milan, Italy; ferrini.f@gmail.com (F.F.); gherlone.enrico@hsr.it (E.G.); cappare.paolo@hsr.it (P.C.); 2 Department of Dentistry, IRCCS San Raffaele Hospital, 20132 Milan, Italy; 3 UniSR-Social.Lab (Research Methods), Faculty of Psychology, Vita-Salute San Raffaele University, 20132 Milan, Italy; pantaleo.giuseppe@hsr.it

**Keywords:** dental implants, dental implant neck design, peri-implant bone loss, peri-implant probing depth

## Abstract

The present study was conducted to investigate whether a different implant neck design could affect survival rate and peri-implant tissue health in a cohort of disease-free partially edentulous patients in the molar–premolar region. The investigation was conducted on 122 dental implants inserted in 97 patients divided into two groups: Group A (rough wide-neck implants) vs. Group B (rough reduced-neck implants). All patients were monitored through clinical and radiological checkups. Survival rate, probing depth, and marginal bone loss were assessed at 12- and 24-month follow-ups. Patients assigned to Group A received 59 implants, while patients assigned to Group B 63. Dental implants were placed by following a delayed loading protocol, and cemented metal–ceramic crowns were delivered to the patients. The survival rates for both Group A and B were acceptable and similar at the two-year follow-up (96.61% vs. 95.82%). Probing depth and marginal bone loss tended to increase over time (*follow-up*: t_1_ = 12 vs. t_2_ = 24 months) in both groups of patients. Probing depth (*p* = 0.015) and bone loss (*p* = 0.001) were significantly lower in Group A (3.01 vs. 3.23 mm and 0.92 vs. 1.06 mm; Group A vs. Group B). Within the limitations of the present study, patients with rough wide-neck implants showed less marginal bone loss and minor probing depth, as compared to rough reduced-neck implants placed in the molar–premolar region. These results might be further replicated through longer-term trials, as well as comparisons between more collar configurations (e.g., straight vs. reduced vs. wide collars).

## 1. Introduction

The scientific debate on dental implant macro-design is a well-known topic in the field of implant dentistry. The ideal fixture design should bring together the most suitable and distinctive characteristics for implant osseointegration, such as type of material (zirconium or titanium), body shape (cylindrical or conical), neck geometry (straight, reduced, or wide), threads depth, width, and pitch, as well as tapered or non-tapered apical portion, body length, and diameter. Although there is no perfect implant design [1,2], nor a best surface treatment [3], scientific evidence has consistently demonstrated that different dental implant macro-designs affect long-term implant success [4,5] and also accelerate the healing process, to allow implant therapy in the population of patients who are more prone to failure [6,7]. Implant collar, being the portion of the implant that connects the fixture with the oral cavity throughout a prosthetic device, is a very important feature related to the peri-implant tissue’s health conditions.

Several studies about implant neck design and marginal bone loss can be found in the literature, but the results are controversial. In vivo animal studies reported a greater crestal bone height and thickness of surrounding implant tissue in dental implants with triangular neck designs [8]; smaller crestal bone loss but similar peri-implant tissue thickness in narrow ring extra-shorts implants [9]; and greater bone loss in dental implants with micro-rings on the neck, as compared to open-thread implant collars [10]. Human model studies reported improved biomechanical behavior for stress/strain distribution pattern in dental implants with divergent collar design [11]; no additional bone loss in non-submerged dental implants with a short smooth collar compared to similar but longer implant collar design [12].

Other clinical findings suggest that specific implant neck design might be suitable in anterior areas, where bone loss, even if acceptable, can lead to adverse aesthetic results [13,14].

The purpose of the present study is to compare peri-implant hard- and soft-tissue health conditions in partially edentulous patients who received the same dental implants but with two different implant neck designs, at a two-year follow-up. In this study, the null hypothesis led to the expectation of no differences in survival rate, probing depth, and marginal bone loss among patients who received dental implants with wide or reduced collar morphology.

## 2. Materials and Methods

### 2.1. Patients

Study participants were selected from patients who attended the Dental Department of IRCCS San Raffaele Hospital, Milan, Italy asking for partial fixed implant-prosthetic rehabilitation. Recruitment occurred from February 2016 to November 2017, and the investigation was conducted following all the ethical regulations related to the institution.

Patients had to meet the following inclusion criteria: (1) hopeless teeth to be extracted at least four months prior to surgery in molar/premolar region; (2) no previous dental implants already in place adjacent to surgical site; (3) natural antagonistic teeth (composite resin restorations allowed); (4) absence of diabetes, periodontitis, bruxism, and smoking; (5) absence of chemotherapy or radiation therapy of head and neck district, as well as anti-resorptive drug therapy (i.e., bisphosphonates); and (6) neither mucosal lesions (lichen planus, epulis fissuratum) nor bone lesions (i.e., simple bone cyst or odontomas). Eligible areas for surgery of edentulous maxilla or mandible were selected to receive 1 to a maximum of 3 dental implants. Participants were verbally informed about the purpose of the study but not assigned to a specific group, as they were randomly chosen either to receive a wide-neck implant (Group A) or a reduced-neck implant (Group B).

Patients were assigned to conditions according to a computer-generated random list, prescribing the use of the reduced vs. wide implant. Clinical measures (i.e., survival rate, peri-implant probing depth, and mean marginal bone loss) were taken at 12 and 24 months. Thus, the design amounted to a 2 (implant: wide vs. reduced) X 2 (time: 12 vs. 24 month follow-up) *mixed* factorial design, following the *Consolidated Standards of Reporting Trials* (CONSORT) guidelines available as supplementary material to this manuscript and on http://www.consort-statement.org/.

Written informed consent was signed before the start of the study; patients were allowed to leave the research at any time, without any consequence.

**Figure 1:**
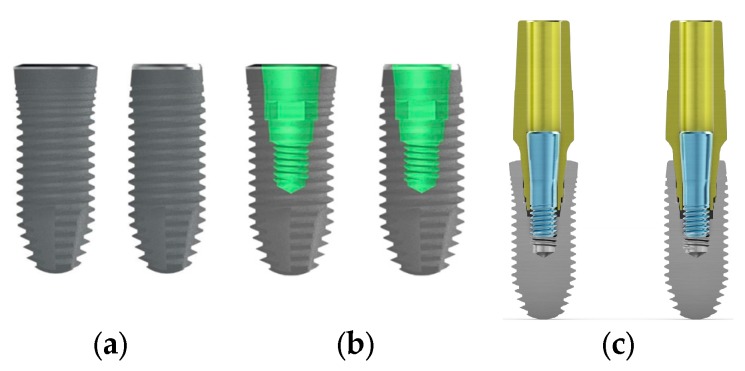
Image shows the CSR full-treatment ZirTi conical dental implant collar with different macro-design. (**a**) Rough wide neck compared with rough reduced neck; (**b**), wide-neck and reduced-neck designs with double conical implant–abutment connection with internal hexagon for prosthetic repositioning; and (**c**) wide-neck and reduced-neck designs with same contact length and tapered angles at the interface.

Implant macrogeometry regarding the two different collar designs used in the present study is shown in Figure 1 (CSR, Sweden & Martina, Due Carrare, Italy).

### 2.2. Implant Surgery

The study was based on a single blind design, with patients being unaware of which type of implant neck design (wide or reduced) was used for the therapy.

Local anesthesia was induced with local infiltration of lidocaine 20 mg/mL with 1:50.000 adrenaline (Ecocain, Molteni Dental, Firenze, Italy). A crestal horizontal incision was made, with buccal relieving incisions in the medial and distal portions of the main incision. A full-thickness flap was raised, and dental implants were placed in edentulous sites of 0.5 mm, subcrestally, with a minimum insertion torque of 35 Ncm. Cover screw was positioned, and a periosteal incision was performed in order to allow flap passivation in search for primary intention healing of the wound. Vertical mattress suturing technique was used with a 4-0 coated braided absorbable suture (Vicryl, ETHICON, Johnson & Johnson, New Brunswick, NJ, USA). Sterile dry gauze compression was performed on the wound to control post-operative bleeding. Ice packages were delivered to the patients immediately after surgery, with instruction to apply cold to the surgical area for the following 24 h. Semi-liquid cold diet was recommended for the first 48 h.

At-home pharmacological therapy prescribed was amoxicillin 1 g, every 12 hours, for six days, and non-steroid anti-inflammatory drug ibuprofen 400 mg, every 12 hours, for four days, post-operatively. All implants were loaded after a 4-month healing period, through a delayed loading protocol, with a composite resin temporary restoration, followed by metal–ceramic cemented crowns. Definitive abutments used for both Group A and B were the same and had conical connection with Double Action Tight (DAT), a system that presents a conical interface between the abutment and the implant, plus one more conical interface between the screw and the abutment.

Clinically, abutment screws were tightened at 25 Ncm by using a dental torque wrench.

### 2.3. Parameters

Dental implant survival rate was defined as the fixtures being osseointegrated and staying in situ; and capable to guarantee stability for prosthetic support along the 2-year observation period following the surgical placement. Peri-implant probing depth was estimated through a CP12 University of North Carolina color-coded periodontal probe (Hu Friedy, Chicago, IL, USA), in the mesial, distal, buccal, and lingual/palatal surfaces of the fixture. Distance in mm between the mucosal margin and the tip of the probe was considered as pocket depth.

Intraoral radiographs were taken, using extension cone paralleling system (XCP, Dentsply international, RINN), and mean marginal bone loss was calculated, using Digora Optime digital intraoral imaging system (Soredex, Tuusula, Finland).

A line was traced parallel to the long axis of the implant in order to measure in mm the distance between the crestal bone level at the margin of the implant neck and the top of the apical portion of the implant.

### 2.4. Statistical Analysis

All analyses were run at the implant level. Peri-implant probing depth and marginal bone loss were submitted to separate 2 (follow-up: t_1_ = 12 vs. t_2_ = 24 months) X 2 (*neck design*: reduced vs. wide) multivariate analyses of variance (MANOVA_s_), in order to distinguish the effects of follow-up time, implant neck design, and additionally assess any interactive effect(s) of the two factors. Mean values were complemented by standard errors of the mean (*Se*) and 95% confidence intervals (CI).

## 3. Results

A total of 97 patients (56 men and 41 women) aged between 33 and 75 years (mean 58.2 ± 6.22 years) were selected for the present study. None of them withdrew from the research, and 122 fixtures were placed in the molar/premolar region.

Fixtures made of titanium grade 4 had a standard length (≥10 mm) and a diameter of 3.8 and 4.2 mm for wide-neck implants and 4.2 and 5.0 mm for reduced-neck ones. Dental implants received the same subtraction procedure, according to the Zir-Ti full-surface treatment (Zirconium Oxide Sand-Blasted and Acid Etched Titanium). The apical portion was tapered with 50° accentuated triangular threads and four longitudinal incisions, to increase penetration ability and anti-rotation features. Fifty patients formed Group A (rough wide-neck design) and received 59 implants. Group B (rough reduced-neck design) was composed of forty-eight patients, who received 63 implants.

**Figure 2:**
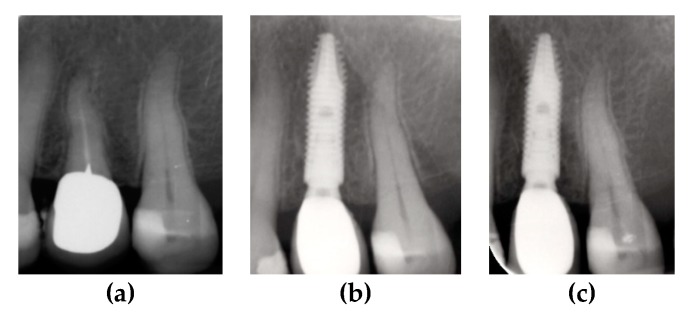
Periapical X-rays showing marginal bone level of CSR dental implant with a reduced neck. (**a**) Pre-operative X-ray; (**b**) post-operative follow-up at 12 months; and (**c**) post-operative follow-up at 24 months.

**Figure 3:**
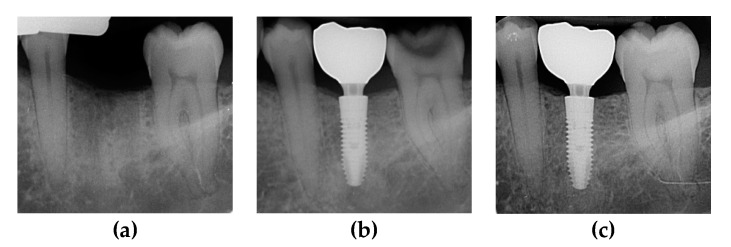
Periapical X-rays showing marginal bone level of CSR dental implant with a wide neck. (**a**) Pre-operative X-ray; (**b**) post-operative follow-up at 12 months; and (**c**) post-operative follow up at 24 months.

The two groups were compared at one-year and two-year follow-ups. Survival rate, probing depth, and marginal bone loss were recorded through clinical and radiological checkups. Radiological records for different dental implants placed in Group A and B patients are shown in Figure 2 and Figure 3.

The overall survival rate of CSR dental implants at the two-year follow-up was 96.72% (four implant failures out of 122 implants placed). Both groups showed similar outcomes: At 12 months, survival rate was 98.30% for Group A and 98.41% for Group B, while it decreased at 96.61% for Group A and 96.82% for Group B at the 24-month follow-up.

Regarding peri-implant probing depth, a 2 (*follow-up*: t_1_ = 12 vs. t_2_ = 24 months) X 2 (*neck design*: reduced vs. wide) multivariate analysis of variance (MANOVA) affirmed a main effect of follow-up, *F* (1, 116) = 10.69, *p* < 0.001, such that probing depth was generally lower at 12 months (3.06 mm ± *Se* = 0.046; 95% CI = 2.96, 3.15) than at 24 months (3.18 mm ± *Se* = 0.050; 95% CI = 3.08, 3.28), independently of type of neck design. Furthermore, the analysis also revealed a main effect of neck design, *F* (1, 116) = 6.28, *p* < 0.015, such that probing depth was generally lower for wide (Group A: 3.01 mm ± *Se* = 0.063; 95% CI = 2.88, 3.13) than for reduced-neck implants (Group B: 3.23 mm ± *Se* = 0.061; 95% CI = 3.11, 3.35), independently of time of follow-up. More specifically, the difference between the two groups, considered at one and two years of follow-up were, respectively, as follows: Group A (one year): 2.93 mm ± *Se* = 0.07; 95% CI = 2.79, 3.07 vs. Group B (one year): 3.18 mm ± *Se* = 0.05; 95% CI = 3.07, 3.28 (*p* = 0.007); and Group A (two years): 3.09 mm ± *Se* = 0.07; 95% CI = 2.95, 3.24 vs. Group B (two years): 3.28 mm ± *Se* = 0.06; 95% CI = 3.15, 3.40 (*p* = 0.061). The interaction *follow-up* (t_1_ = 12 vs. t_2_ = 24 months) X *neck design* (reduced vs. wide) was not significant, *F* (1, 116) = 0.58, *p* = 0.45, n.s.

A 2 (*follow-up*: t_1_ = 12 vs. t_2_ = 24 months) X 2 (*neck design*: reduced vs. wide) multivariate analysis of variance (MANOVA) was also conducted for marginal bone loss and revealed a main effect of follow-up, *F* (1, 116) = 198.85, *p* < 0.001, such that marginal bone loss was generally lower at 12 months (0.89 mm ± *Se* = 0.02; 95% CI = 0.86, 0.93) than at 24 months (1.08 mm ± *Se* = 0.01; 95% CI = 1.06, 1.11), independently of type of neck design. Furthermore, the analysis also revealed a main effect of neck design, *F* (1, 116) = 34.04, *p* < 0.001, such that marginal bone loss was generally lower for wide (Group A: 0.92 mm ± *Se* = 0.02; 95% CI = 0.88, 0.95) than for reduced-neck implants (Group B: 1.06 mm ± *Se* = 0.02; 95% CI = 1.03, 1.10), independently of time of follow-up. More specifically, the difference between the two groups, considered at one and two years of follow-up, were, respectively, as follows: Group A (one year): 0.84 mm ± *Se* = 0.03; 95% CI = 0.78, 0.88 vs. Group B (one year): 0.95 mm ± *Se* = 0.02; 95% CI = 0.91, 0.99 (*p* = 0.001); and Group A (two years): 1.00 mm ± *Se* = 0.02; 95% CI = 0.97, 1.03 vs. Group B (two years): 1.17 mm ± *Se* = 0.02; 95% CI = 1.14, 1.20 (*p* = 0.001). Importantly, the two-way interaction *follow-up* (t_1_ = 12 vs. t_2_ = 24 months) X *neck design* (reduced vs. wide) was statistically significant, *F* (1, 116) = 3.91, *p* = 0.05, showing that the increase in bone loss for reduced-neck implants (Group B) was steeper than the increase observed for wide-neck implants.

## 4. Discussion

Our study focused on dental implants’ macro-design, particularly on the clinical performance of the same type of fixture but with two different rough collar designs in partially edentulous patients, using a delayed loading protocol. Examined parameters were peri-implant probing depth, marginal bone loss, and survival rate at two-year follow-up. Both groups of patients showed an acceptable but almost similar implant survival rate. However, patients who received implants with a wide-neck design presented lower probing depth and minor marginal bone loss compared to reduced neck; thus, the null hypothesis of no differences between dental implants with different neck designs was partially rejected. From a clinical point of view, differences in probing depth and marginal bone loss between Group A and B were not relevant at the two-year follow-up. Since the absence of signs of soft-tissue inflammation and the absence of further additional bone loss following initial healing were found, according to peri-implant health definition by Renvert et al. [15], it can be affirmed that both groups of patients showed peri-implant tissue health conditions.

Implant therapy is a very helpful discipline when it comes to rehabilitating dental patients. Even if bone loss around oral implants is described to be an unavoidable and physiologic foreign-body reaction of bone against titanium [16,17,18], the key for success resides in the neutralization of risk factors at multiple levels: patient level, implant level, and prosthetic level.

Risk factors such as diabetes, periodontitis, bruxism, smoking, antidepressants intake, bone augmentation procedures, head and neck radiotherapy [19,20,21,22] play a principal role in long-term implants’ outcome. These factors are found at the patient level, meaning that they are poorly controllable over time, as they can worsen along with local or systemic health conditions. Here, we must recall that patients included in the present study where disease-free individuals.

Other factors that are set at prosthesis level also interfere with the success of implant therapy and should not be underestimated. According to Vazquez-Alvarez et al. [23], the distance between the implant platform and the horizontal component of the prosthesis has a significant influence on peri-implant bone loss, and to be adequate, it should range from 3.3 to 6 mm. According to Lemos et al. [24], the retention system for implant-supported prostheses may lead to a different bone-loss pattern, as cement-retained restorations showed less marginal bone loss than screw-retained restorations, and implant survival rate was in favor of cement-retained prosthesis.

Restorations for the present study were cemented crowns where a minimum distance of 3.5 mm was kept between implant-abutment junction and horizontal prosthetic component, and where extreme attention was payed to remove any cement excess that could be found underneath them.

Accuracy of dental impression used, whether traditionally or digitally taken, may lead to differences in the fit of the definitive restoration [25]. In our case, prosthetic rehabilitations were performed by passing through light and putty consistency polyvinylsiloxane materials.

The type of prosthetic material itself is described to be capable of having an effect on the peri-implant tissues [26]. In this study, the decision for metal–ceramic crowns was supported by appropriate biomechanical properties, as it was demonstrated in the literature [27,28,29].

Occlusal forces were exerted against natural antagonistic teeth in the molar/premolar region, to standardize the procedure and avoid contact with previously installed dental restorations made of unknown or undefined material properties (e.g., a preexisting zirconium-based bridge in the antagonistic region).

Finally, implant therapy risk factors are also found at the implant level, being the fixture macro-design capable to affect the osseointegration process, as reported by several authors [4,5,30,31,32,33]. Fixture micro- and macro-designs can be adequately selected before treatment, and with the ideal concept design, implant success rate would be more predictable.

Starting from the type of material from which implants are manufactured, different osseointegration processes (amount of bone attachment to the surface and strength of the bone-surface interaction) may occur at the bone level.

Recently reported by Taek-Ka et al. [34], a qualitative different osseointegration was found through higher bone-surface interaction in commercially pure titanium grade 2 implants compared to grade 4. Apart from titanium, zirconia has also been proposed as an alternative material for oral fixtures. At the moment, despite its optimal biocompatibility, no definitive decision is available on the clinical performance of such implants [35,36].

Back to implant collar, the manner in which it is configured appears to be of relevant interest: The maximum loading stress distribution in bone is localized at the neck of the implants, as described by Anitua et al. [37] and Huang et al. [38]. Several studies are available in the literature, but no consensus on which collar design is more suitable for osseointegration was agreed on by the authors.

Our study would qualify rough wide-neck implants to reduce bone loss over time, being conscious that a longer follow-up period is necessary to confirm these findings. This may be related to the platform-switching concept, which has been described to be beneficial for osseointegration [39,40,41,42,43]. In fact, even in the case that a platform-matched abutment is used in such implants, a minimal effect of switching platform still exists, being that the neck of the implant is wider in diameter with respect to the main body. Otherwise, reduced-neck implants are less likely to benefit from the platform-switching effect because of their narrower platform.

According to Eshkol-Yogev et al. [44], round neck implants may significantly increase primary stability when compared to triangular neck design. In a paper by Mendoca et al. [45], bone remodeling showed to be of benefit around implants with rough collar design, in mandible but not in maxilla, if compared to machined collar surface implants. In a review by Koodaryan et al. [46], rough-surfaced micro-threaded neck implants appeared to lose less bone compared to polished and rough-surfaced neck implants.

CSR implants placed in this study had roughened surface collars with no microthreads at the bone cervical region. Presence or absence of microthreads, as well as the amount of surface roughness, may have an effect on bone preservation. Despite that an implant collar with a microthread can help in the maintenance of peri-implant bone against prosthetic loading, [47] this study was focused on conventional rough-surface dental implants, not to add confounding aspects related to numerous available surface topography (e.g., smooth, polished neck vs. machined surface vs. microthread design). Furthermore, CSR implants had a moderate degree of roughness, as no beneficial effect seemed to be associated with an increase in surface roughness. In fact, a 20-year follow-up clinical trial by Donati et al. [48] reported no peri-implant bone preservation related to implants with an increased surface roughness.

Another relevant issue to consider is the implant–abutment connection system. Implants in the present study were provided with DAT connection. Consisting of a double conical interface and internal hexagon for prosthetic repositioning, this type of connection follows the recent literature’s outcomes. According to Caricasulo et al. [49], internal connection, particularly conical interfaces seem to better maintain crestal bone level around dental implants.

As stated by Kim et al. [50], transmission of the occlusal load from the restoration to the implant, and then from the implant to the surrounding bone, is essential to stimulate osteoblasts activity. This is to say, to avoid minimum but regular and continuous bone resorption, described to be around 1 mm for the first year and of 0.2 mm per year thereafter [51], bone deposition must be encouraged.

The concept of biocompatibility related to implant-prosthetic rehabilitation can be considered as the ultimate key for success: proper design of the fixture, together with a correct function of the implant–abutment connection, and optimal adaptation of the prosthetic restoration generates a self-defensive mechanism that guarantees long-term survival rates.

Considering multiple and confounding aspects which affect implant failure, with risk factors set at patient, implant, and prosthetic level, it is important to affirm that bone loss in not solely determined by collar morphology. Further studies should be conducted on multiple heterogeneous implant collar design in different populations (e.g., diabetic vs. nondiabetic) and with different prosthetic restorations (e.g., screwed vs. cemented). Longer follow-up periods could highlight the enhancement of the clinical performance of dental implants with specific neck configurations.

## 5. Conclusions

Within the limitations of the present prospective clinical comparative study, peri-implant probing depth and marginal bone level around dental implants placed in edentulous sites in molar/premolar region were affected by different neck designs. Patients who received implants with rough wide-neck design presented lower probing depth and minor marginal bone loss compared to patients with rough reduced-neck implants.

Reduced-neck implants showed a tendency to lose comparatively more bone over time if compared with wide-neck implants.

However, dental implants’ survival rate was acceptable and satisfactory for both groups of patients and showed no differences at the two-year follow-up.

## Supplementary Materials

The following are available online at https://www.mdpi.com/1996-1944/13/5/1029/s1, Consort Statement and 2010 Checklist.
